# Self-guided retrospective motion correction (SEGMO) for free-breathing whole-heart coronary MRA with 100% acquisition efficiency

**DOI:** 10.1186/1532-429X-14-S1-M10

**Published:** 2012-02-01

**Authors:** Jianing Pang, Himanshu Bhat, Behzad Sharif, Zhaoyang Fan, Edward B Gill, James Min, Troy Labounty, Louise Thomson, John D Friedman, Daniel S Berman, Debiao Li

**Affiliations:** 1 Departments of Radiology and Biomedical Engineering, Northwestern University, Chicago, IL, USA; 2 Biomedical Imaging Research Institute, Cedars Sinai Medical Center, Los Angeles, CA, USA; 3 Siemens Medical Solutions USA Inc., Charlestown, MA, USA

## Summary

A respiratory motion correction method (SEGMO) is proposed for whole-heart coronary MRA. It eliminates the need for a diaphragm navigator, reduces imaging setup time, and has more accurate respiratory motion detection using an affine model. Its inherent 100% gating efficiency ensures a shorter and more fixed scan time compared to conventional navigator gated schemes.

## Background

Free-breathing whole heart coronary MRA uses diaphragm navigator to gate data acquisition, which suffers from the need for time-consuming and exquisite positioning, prolonged scan time due to low gating efficiency, and inaccuracy in motion detection. In this work, a respiratory motion correction scheme is proposed with an affine motion model for accurate estimation of respiratory motion, and uses 1D projections to derive heart position and segment 3D radial data into respiratory bins.

## Methods

A dual-echo self-guiding module is added prior to each imaging segment to suppress static chest wall [1]. Respiratory superior-inferior translations are derived from 1D heart profiles using a cross-correlation method, which are subsequently used to segment data into respiratory bins similar to the work in [2]. From each bin a low-resolution image is reconstructed. Next, to estimate the motion in the image domain, affine motion registration is performed between each moving bin and an end-expiratory reference bin, followed by motion correction using the estimated affine parameters. Finally, all corrected k-space data is combined to give a high-resolution image free of motion artifacts.

Twelve healthy volunteer scans were performed on a clinical 1.5T scanner (MAGNETOM Espree, Siemens AG Healthcare, Erlangen, Germany) with IRB approval and written consents. MR data was acquired using an ECG-gated, T2-prepared, fat-saturated SSFP pulse sequence with 3DPR trajectory (TR/TE=3.2ms/1.6ms, FOV=260mm^3^, matrix size=256^3^, voxel size=1.0mm^3^, flip angle=90°, readout bandwidth=781Hz/pixel, total number of views=16000 to 16800,Scan time=7.6±1.5min). Three 3D images were reconstructed from each dataset: one without motion correction (NO), one corrected with navigator binning (NAV), and one with the proposed self-guided binning (SEGMO). Diaphragmatic navigators were set up but were only used in the NAV reconstruction (not SEGMO). Images were reformatted using CoronaViz software (Siemens Corporate Research, Princeton, NJ).

## Results

Quantitative comparisons of LAD, LCX and RCA length and sharpness are performed between the three reconstructions. Both SEGMO and NAV perform better (p<0.05) than NO, and show no significant difference between each other. Qualitative image scoring yielded similar results. Numbers are summarized in Table 1.

**Table 1:** Paired t-test results from comparing the three reconstructions. Statistically significant results are in bold and italic (p<0.05)

	LCX				LAD				RCA				Overall image quality score
	Length	Sharpness	Max diameter	Min diameter	Length	Sharpness	Max diameter	Min diameter	Length	Sharpness	Max diameter	Min diameter	

NO, NAV	***0.0008***	***0.0051***	0.3554	0.4145	***0.0006***	***0.0001***	0.7818	0.4781	***0.0063***	***0.0263***	0.7090	0.6178	***0.0000***
NO, SEGMO	***0.0012***	***0.0233***	0.3793	0.9711	***0.0008***	***0.0008***	0.7712	0.8985	***0.0071***	***0.0231***	0.3291	0.9647	***0.0000***
NAV, SEGMO	0.5950	0.1687	0.9136	0.3436	0.7087	0.7997	0.1631	0.3347	0.3144	0.4841	0.4008	0.6504	0.4678

## Conclusions

The proposed motion correction method eliminates the need for a diaphragm navigator, reduces imaging setup time, and is more accurate in respiratory motion detection. The inherent 100% gating efficiency ensures a shorter and more fixed scan time compared to conventional navigator gated schemes. The affine model provides realistic motion estimation and thus minimizes residual motion artifacts. The current reconstruction time is ~two hours. However, parallel computing and code optimization will greatly accelerate the reconstruction and hence will make the method feasible for clinical practice.

## Funding

National Institute of Health grants nos. NIBIB EB002623 and NHLBI HL38698.

**Figure 1:**
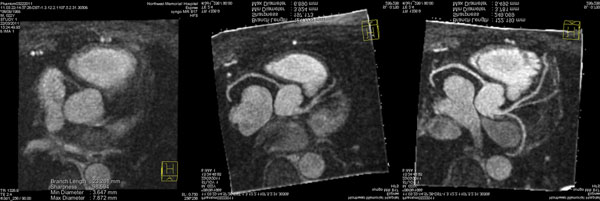
Reformatted image without correction (left), with navigator binning (middle), and with SEGMO (right).
